# The effect of high-intensity interval training on type 2 diabetic muscle: A metabolomics-based study

**DOI:** 10.1016/j.heliyon.2024.e34917

**Published:** 2024-07-20

**Authors:** Kayvan Khoramipour, Mohammad Amin Rajizadeh, Ziba Akbari, Mohammad Arjmand

**Affiliations:** a Endocrinology and Metabolism Research Center, Kerman University of Medical Sciences Kerman, Iran; b i+HeALTH Strategic Research Group, Department of Health Sciences, Miguel de Cervantes European University (UEMC), 47012 Valladolid, Spain; c Physiology Research Center, Institute of Neuropharmacology, Kerman University of Medical Sciences, Kerman, Iran; d Metabolomics Lab, Department of Biochemistry, Pasteur Institute of Iran, Tehran, Iran

**Keywords:** Type 2 diabetes, High-intensity interval training, Muscle, Rat, Metabolomics

## Abstract

**Background:**

This study aimed to investigate the effect of eight weeks of high-intensity interval training (HIIT) on muscle metabolism in rats with type 2 diabetes (T2D) using metabolomics approaches.

**Methods:**

20 male Wistar rats at the age of 8 weeks-were assigned to four groups of five, each in the group randomly: control (CTL), type 2 diabetes (DB), HIIT (EX), and type 2 diabetes + HIIT (DBX). T2D was induced by two months of a high-fat diet plus a single dose of streptozotocin (35 mg/kg). Rats in the EX and DBX groups performed eight weeks of HIIT (running at 80–100 % of Vmax, 4–10 intervals). NMR spectroscopy was used to determine the changes in the muscle metabolome profile after training.

**Results:**

Changes in metabolite abundance following exercise revealed distinct clustering in multivariate analysis. The essential metabolite changes between the DB and CTL groups were arginine metabolism, purine metabolism, phosphate pathway, amino sugar metabolism, glutathione metabolism, and aminoacyl-tRNA biosynthesis. However, Arginine biosynthesis, pyrimidine metabolism, aminoacyl-tRNA biosynthesis, and alanine, aspartate, and glutamate metabolism were altered between the DBX and DB groups.

**Conclusion:**

These results suggest that eight weeks of HIIT could reverse metabolic changes induced by T2D in rat muscles, contributing to reduced FBG and HOMA-IR levels.

## 1 Introduction

Diabetes represents a chronic condition characterized by elevated blood glucose levels resulting from insufficient insulin production or impaired insulin action [1,2]. Type 2 diabetes (T2D), which accounts for approximately 90 % of all cases, is characterized by insulin resistance and inadequate insulin secretion [[3], [4], [5]]. The global prevalence of T2D is projected to significantly escalate from 463 million cases in 2019 to over 700 million cases by 2045, leading to an associated increase in annual costs from $760 billion to approximately $845 billion during this period [3,6]. Unmanaged diabetes can lead to severe eye, kidney, nerve, heart, and muscle complications, escalating costs [1,6].

In individuals with type 2 diabetes, muscle tissues exhibit metabolic alterations, particularly glucose uptake and utilization [7,8]. This results in a reduced availability of glucose fuel for muscle growth and development. Inadequate insulin function causes increased protein breakdown, which leads to muscle wasting. Moreover, diabetes has been linked to impaired mitochondrial function in skeletal muscle [9], reducing metabolic flexibility, interfering with signaling pathways, and exacerbating insulin resistance. Increased oxidative stress also contributes to insulin resistance in muscle tissue [10,11].

Conversely, the muscle tissue is highly responsive to exercise, which is crucial in managing metabolic disorders such as diabetes. Skeletal muscles play a pivotal role in regulating energy homeostasis and consumption. Research indicates that exercise enhances glucose uptake, glycolysis, glucose oxidation, intra-muscular triglyceride (IMTG) hydrolysis, turnover, and fat oxidation in the muscle [12,13].

While the benefit of physical activity/exercise on glycemic control in T2D has been established, time constraints are a commonly reported barrier to exercise [14]. High-intensity interval training (HIIT) has garnered attention recently as a time-efficient means of improving glycemic control and cardiovascular health in patients with T2D [15]. In comparison to other continuous high-intensity exercise options, HIIT has been reported to be enjoyable for some despite a stronger feeling of fatigue [16]. HIIT is considered an alternating vigorous-intensity exercise (aerobic or strength training) with recovery stages that can be either resting or low-intensity exercise [16].

HIIT has been demonstrated to improve glycemic control [17,18]. The effects of HIIT on blood glucose could be partly mediated by improved skeletal muscle mitochondrial function [17,19].

As with any exercise program, the initial fitness level should be considered so that exercise acclimation periods are appropriate and that the inclusion of warmups and cool-downs is warranted [16]. An additional risk associated with high-intensity training is exercise-related hypoglycemia, particularly among insulin users. However, non-insulin users (e.g., T2D) have minimal risk and would benefit from HIIT to maintain their glycemic status [20].

Prior studies have employed techniques to measure gene and protein responses to exercise or exercise training [21,22]. However, comprehensive analysis of metabolites, the end products of intricate interactions within and outside the cell, offers valuable insights into the interactions between genes and the environment during exercise [23,24]. Metabolomics, the study of changes in metabolites, thus presents an ideal approach for assessing phenotype and physiology in exercise science, particularly within muscle tissues [25].

Utilizing metabolomics, Xiang et al. explored the effects of five weeks of endurance exercise on muscle tissue in T2D rats. The results demonstrated significant alterations in 95 metabolites in exercised diabetic rats, correlating with improved insulin resistance and reduced T2D-related complications. Nevertheless, no study has employed metabolomics to investigate the effect of high-intensity interval training (HIIT) on muscle metabolism in diabetes [26]. HIIT presents a promising solution to time constraints that hinder regular exercise and could lead to more profound metabolic adaptations in skeletal muscles [27]. Thus, our study aimed to investigate the effects of eight weeks of HIIT on the muscle metabolome profile in diabetic rats.

## 2 Methods

### 2.1 Animals

**Fig. 1:**
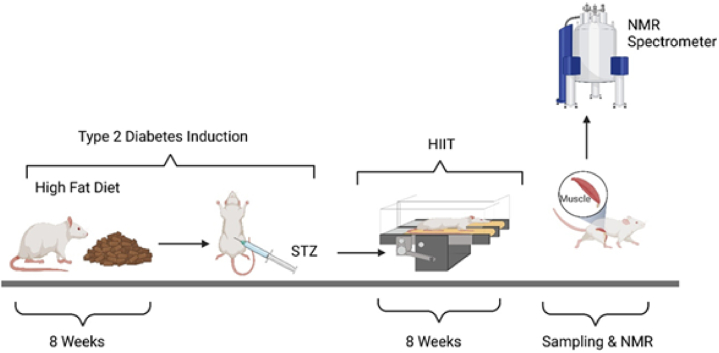
Timing of study.

Twenty male Wistar rats, aged eight weeks with an average weight of 200 g, were acquired from the Kerman University of Medical Sciences Animal Farm. The rats were housed in polycarbonate cages under controlled environmental conditions (average temperature, 22 ± 1.4 °C, humidity, 50 ± 4 %; 12:12 light-dark cycle). The ethical guidelines set by the Kerman University of Medical Sciences ethics committee (Ethics code: IR. KMU.REC.1399.688, approval on: 10/3/2021) were strictly followed for all animal-related procedures, including housing and euthanasia. The rats were randomly divided into four groups of five each: Diabetes Exercise (DBX), exercise training (EX), Diabetes Control (DB), and Healthy Control (CTL) (Fig. 1).

### 2.2 Induction of type 2 diabetes

The diabetic groups were fed a high-fat diet (HFD) for two months. The ingredients of HFD are listed in Table 1. HFD was purchased from Zist Royan Co., Isfahan, Iran. Following a 12-h fasting period, the rats received a single intraperitoneal dose of streptozotocin (STZ) (35 mg/kg). Three days after the STZ injection, fasting blood glucose (FBG) levels were measured using a glucometer (Accu-Chek, USA). Rats with fasting blood glucose levels of >300 mg/dL were considered diabetic. FBG levels were also measured at baseline (month 0), after diabetes induction (month 2), and after the training protocol in all groups (month 4) [28].

**Table 1:** High-fat and regular diet ingredients.

Diet ingredients	Fat	Carbohydrate	Protein	Fiber/Mineral/Vitamin	Total energy
**Regular diet**	10 %	70 %	19 %	1 %	341 Cal/100 g or 1432 J/100 g
**High-fat diet**	60 %	20 %	19 %	1 %	429 Cal/100 g or 1802 J/g

### 2.3 Exercise protocol

The exercise protocol utilized was the K1 protocol, as previously described [[28], [29], [30], [31], [32], [33], [34], [35], [36], [37], [38], [39], [40]]. Ex and T2D + EX rats underwent a five-day acclimatization period on a treadmill. Subsequently, an incremental running test was conducted to determine the maximum speed (Vmax) before starting the exercise protocol and every two weeks during the protocol. The HIIT program, lasting eight weeks, was designed based on Vmax (80–100 % Vmax, 4–10 intervals) [35,36,41].

### 2.4 Sample collection

Forty-eight hours after the final training session, rats were euthanized by intraperitoneal injection of ketamine (80 mg/kg) and xylazine (10 mg/kg). Blood samples were drawn from the heart, and the soleus muscle was removed and washed with saline. Blood samples were stored in a refrigerator for 30 min and then centrifuged at 1000 g for 20 min at 4 °C. The resulting serum and muscle samples were stored at −80 °C.

### 2.5 Insulin and insulin resistance assessment

Serum insulin levels were measured using the appropriate kits (Rat ELISA Kit, Eastbiopharm). Homeostasis model assessment (HOMA) was used to assess insulin resistance (HOMA-IR) using the following formula: HOMA-IR = [(fasting glucose (mmol/L) × fasting insulin (μU/mL))/22.5].

### 2.6 Muscle cell extraction

The muscle tissue was weighed and ground to a fine powder in liquid nitrogen using a mortar and pestle. Ice-cold 12 % perchloric acid (4 °C) was added (3 ml/g tissue) to halt the metabolism. The samples were vortexed, pulse-sonicated, and centrifuged at 13000 rpm for 20 min at 4 °C. The supernatant was collected, and the pellet was re-extracted with perchloric acid. The supernatant was neutralized with 1 M NaOH to adjust the pH, and the precipitated salt was removed by centrifugation. The resulting supernatant was lyophilized for further analysis.

### 2.7 NMR spectroscopy

Lyophilized samples were dissolved in 100 mM phosphate buffer (pH 7.0) prepared in D2O, with one mM trimethylsilyl propanoic acid (TSP) as an internal reference and 2 mM imidazole as a pH indicator. After centrifugation at 10000 rpm for 10 min at 4 °C, 500 μl of the extracted samples was transferred to NMR probes. NMR spectra were recorded using a 400 MHz Bruker NMR spectrometer with a field gradient operating at 400.13 MHz for proton observation at 298 K with 650 scans. One-dimensional 1H NMR spectra were obtained using a standard 1D NOESY pulse sequence to suppress the residual water peak during spectrum acquisition.

### 2.8 Data analysis

#### 2.8.1 Multivariate analysis

Spectral preprocessing was performed in the MATLAB package (version 7.8.0.347) using the ProMetab (v. 3) function file. This preprocessing step encompasses a range of procedures, including baseline correction, noise reduction, normalization, and scaling, to ensure data quality. Subsequently, each spectrum was aligned and binned at 0.005 ppm, facilitating further analysis by grouping similar chemical shifts and reducing the data complexity. To minimize interference from water signals, the peak at 4.7 ppm (representing water) was removed from all spectra because water signals can dominate the spectra and hinder the accurate analysis of other metabolites. Peaks concerning imidazole at 7.15 and 7.73 ppm were also removed.

The classification method Partial Least Squares Discriminant Analysis (PLS-DA) was employed for multivariate data analysis. PLS-DA is a potent statistical technique that can discern patterns and relationships within intricate datasets.

The outcomes of PLS-DA analysis were conveyed via score plots, loading plots, and variable importance in projection (VIP) plots. Chemical shifts projected above a VIP score of 2.0 were cross-referenced with the human metabolome database (HMDB) and biological magnetic resonance data bank (BMRB) to detect outlier metabolites.

Classified data were cross-validated by 5-fold cross-validation methods, and accuracy was ensured by using false positive (R2) and false negative (Q2) methods to ensure that no data was overfitted. Q2 is an estimate of the model's predictive ability.

There are many methods for analyzing pathways; over-representation analysis (ORA) is one such method that works by identifying pathways or metabolite sets that have a higher overlap with a set of molecules of interest than expected by chance. Therefore, the result metabolite was taken in pathway analysis modules, and pathway enrichment analysis and pathway topology analysis methods were performed using MetaboAnalyst (v.5). A pathway impact value threshold of >0.05, and -log(p) > 1.5 was taken to determine whether a pathway was significantly impacted in our investigation. Pathway impact is a combination of centrality and pathway enrichment results. It is calculated by adding the importance measures of each matched metabolite and then dividing by the sum of the importance measures of all metabolites in each pathway.

#### 2.8.2 Statistical analysis

Fasting Blood Glucose (FBG) levels were analyzed using a two-way analysis of variance (ANOVA). In contrast, insulin and HOMA-IR (Homeostatic Model Assessment of Insulin Resistance (HOMA-IR) were analyzed using one-way ANOVA. Post-hoc testing was performed using Tukey's method. The significance level for all analyses was set at p < 0.05.

## 3 Results

### 3.1 FBG and HOMA-IR

**Fig. 2:**
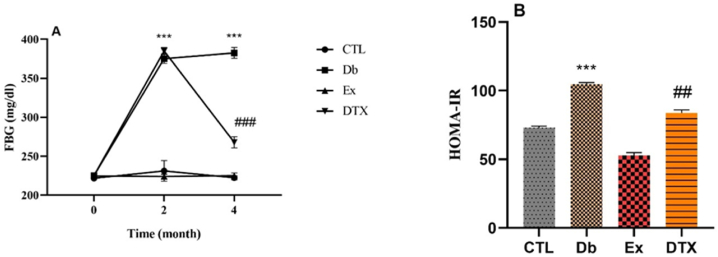
Effects of diabetes and HIIT on FBG and HOMA-IR levels. CTL (control), Db (diabetes), DTX (diabetes + exercise). ***P < 0.001 compared to the CTL group. ##P < 0.01 & ###P < 0.001 compared to the Db.

The results showed a significant increase in FBG levels after diabetes induction (month 2) compared to the baseline (month 0) in both the DB and DTX groups (P < 0.001), with no difference between these groups. HIIT also reduced FBG levels (4 months vs. 2 months) (P < 0.001). We assessed the insulin sensitivity index (HOMA-IR) to determine whether exercise improved insulin sensitivity. Our findings indicated that HOMA-IR increased in the Db group compared to the CTL group (P < 0.001). HOMA-IR was lower in the DTX group than in the DB group (P < 0.01) (Fig. 2). In contrast, body weight significantly increased in the Db and DTX groups after diabetes induction (month 2) in rats. In addition, the weight decreased in the Db and DTX groups, with a more significant decrease in the Db group (P < 0.05) in the post-test (month 4). (Table 2).

**Table 2:** Body weight (BW).

Parameter	Group	Before T2D Induction (Month 0)	After T2D Induction (Month 2)	After Training (Month 4)
BW (gr)	CTL	214 ± 18	285 ± 14^a^	317 ± 13
	Db	205 ± 12	398 ± 25**	307 ± 15^b^
	EX	211 ± 9	283 ± 11^a^	314 ± 8
	DTX	202 ± 10	381 ± 21**	319 ± 21^b^

(Mean ± SEM) before starting the intervention (month 0), after diabetes induction (2 months of high-fat diet and STZ injection) (month 2), and 48 h after the last training session (month 4) in all groups.

a Significant difference at month 0.

b Significant difference at month 2. CTL (control), Db (diabetes), EX (exercise), and DBX (diabetes + exercise).

### 3.2 Multivariate data analysis

**Fig. 3:**
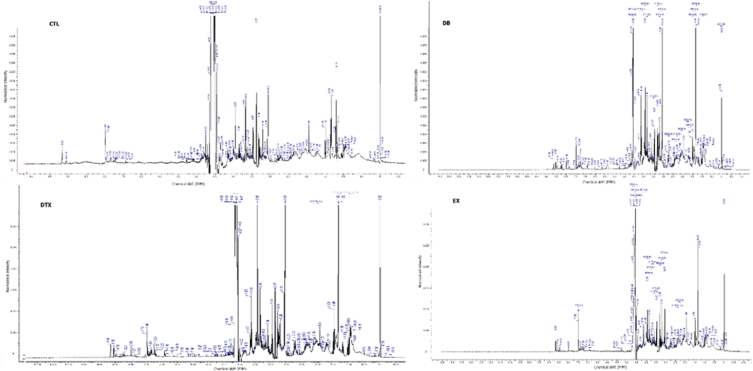
Effects of diabetes and HIIT on NMR Spectra. Control (CTL), Diabetes (DB), Diabetes + exercise (DTX), and Exercise group NMR Spectra's.

**Fig. 4:**
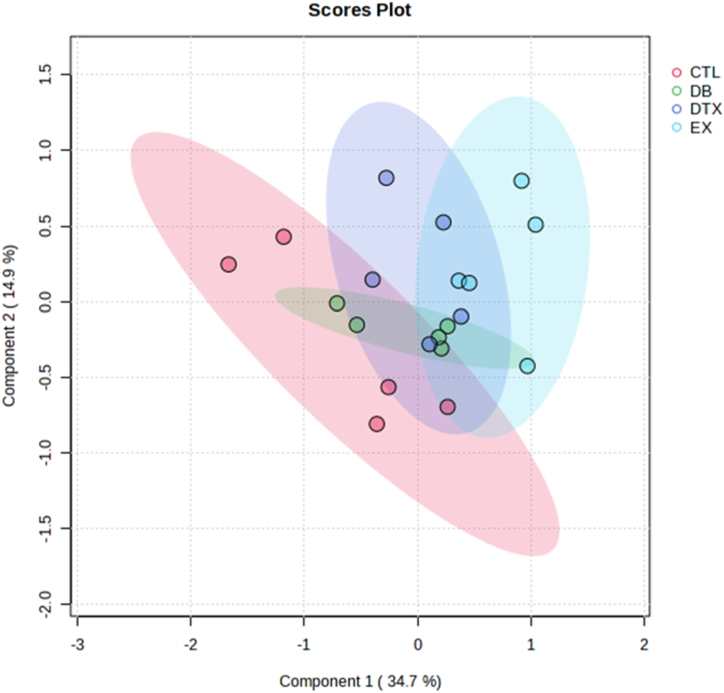
Effects of diabetes and HIIT on the score plot of the selected components. Control (CTL), Diabetes (DB), Diabetes and exercise (DTX), and Exercise group (EX).

**Fig. 5:**
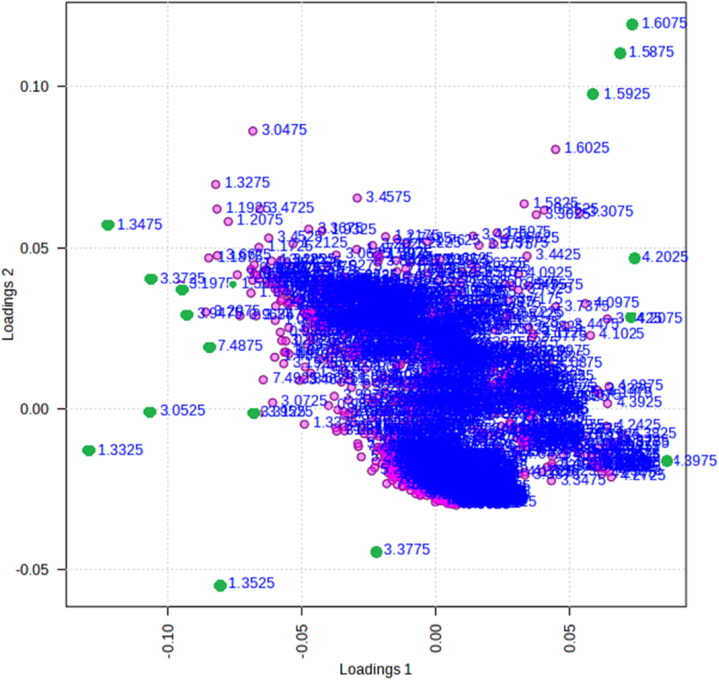
Effects of diabetes and HIIT on PLS-DA loading plot based on 1H NMR data. The spots represent chemical shift outliers among the four study groups. Green circles indicate the variables corresponding to the first 15 VIP outliers.

**Fig. 6:**
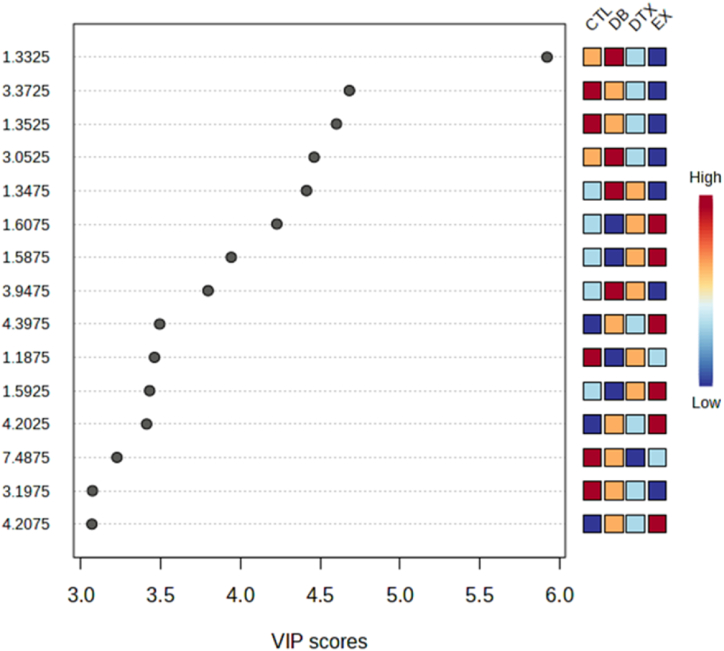
Effects of diabetes and HIIT on the VIP scores. Control (CTL), diabetes (DB), diabetes + exercise (DTX), and exercise group (EX) variable concentrations are shown in the column, where the red color signifies an increase in the variable concentration. In contrast, the blue color indicates a decrease in the variable concentration. The y-axis variable represents the metabolite's 1H NMR chemical shift. The x-axis shows Variable VIP scores used for analysis.

The NMR spectra of all groups are shown in Fig. 3, and the PLS-DA classification score and loading plots are shown in (Fig. 4, Fig. 5). After comparing, the group chemical shift outlier's projection VIP score was calculated and shown in Fig. 6.

VIP chemical shifts were matched in the HMDB database, and the corresponding metabolites were determined (Table 3).

**Table 3:** The list of converted metabolite outliers.

Metabolites	Metabolites	Metabolites
Decamethonium	Glucosamine 6-phosphate	Argininosuccinic acid
Inosinic acid	d-Methionine	l-Methionine
Adenosine monophosphate	Erythritol	*N*-Acetyl-d-glucosamine
d-Xylulose	Ribothymidine	Choline
*N*-Acetyl-l-aspartic acid	2-Hydroxyphenethylamine	Isovaleric-acid
Shikimic acid	Chlorogenic acid	l-Acetylcarnitine
Galactonolactone	Aminocaproic acid	Pyridoxamine
NADP 1	Hippuric acid	D-Glyceraldehyde 3-phosphate
NAD	Glutaconic acid	Glycerol 3-phosphate
Biotin	butanol 1-Butanol	S-Adenosylhomocysteine
2-Methylglutaric acid	Hydroxyoctanoic acid	Propane-1,3-diol
Spermine	Monoethyl malonic acid	Leucinic acid
Serotonin	O-Acetylserine	l-Homoserine
Galacturonic acid	l-Threonine	FAD
Pantothenol	Sucrose	Thiamine
Fucose l-Fucose	Neostigmine	d-Mannose
3-Aminoisobutanoic acid	6-Aminopenicillanic acid	l-Tyrosine
l-Threonine	1-Kestose	Cytidine
2-Fucosyllactose	Lacto-*N*-tetraose	Alpha-d-Glucose 1,6-bisphosphate
NADPH	Uridine diphosphate glucuronic	Inosine
d-Xylose	5-Phenylvaleric acid	Scyllo-Inositol
l-Isoleucine	l-Gulonolactone	L-Threitol Erythritol
Malic acid	Levoglucosan	Dihydroxyacetone
l-Serine	l-Norleucine	Pipecolic acid
L-Tryptophan	Indoleacetic acid	L-Histidinol
3-Hydroxybutyric acid	dADP	Isoferulic acid
Pectic acid	l-Cysteine	D-*threo*-Isocitric acid
D-Alanyl-d-alanine	Folic acid	l-Proline
2-Amino-2-deoxy-d-gluconate	Cyclohexylamine	Homovanillic acid
Hypotaurine	1-Methylhistidine	Tryptamine
Phenylacetyl glycine	*N*-Acetylputrescine	5′-triphosphate dGTP
Nicotine	O-Succinyl-l-homoserine	Melibiose
Cellobiose	l-Lactic acid	Ethylmalonic acid
Ureidopropionic acid	LNnT Neolactotetraose 2	Lactose
Lactulose	l-Lactic acid	HEPES Gibberellin A67
Arecoline	Cotinine	3,5-Diiodo-l-tyrosine
l-Arabinose	Homo-l-arginine	O-Phosphoethanolamine
Serine	d-Tagatose	2-Hydroxycaproic acid
5′-monophosphate	Cytosine	cholic acid Cholic acid
epsilon-Caprolactam	d-Ribulose 5-phosphate	Fructose 6-phosphate
Glycylproline	*N*-Acetylglutamic acid	l-Cystathionine
Adonitol Ribitol	NADH	d-Ribose 5-phosphate
Uridine triphosphate	D-Pinitol	Acetylcholine
Putrescine	Histamine	Uridine 5′-diphosphate
Spermidine	Inosine triphosphate	CDP
Guanosine diphosphate	ADP	2-Chloroethanol
Methionine sulfoxide	Ornithine	Hydrochloride Spectinomycin
Ureidosuccinic acid	d-Fructose 2,6-bisphosphate	Cytidine triphosphate
d-Ornithine	*N*-Acetylmannosamine	Guanosine
Cytidine monophosphate	d-Fructose	Methylmalonic acid
Atropine	D-Glyceraldehyde	

### 3.3 pathway analysis result

**Fig. 7:**
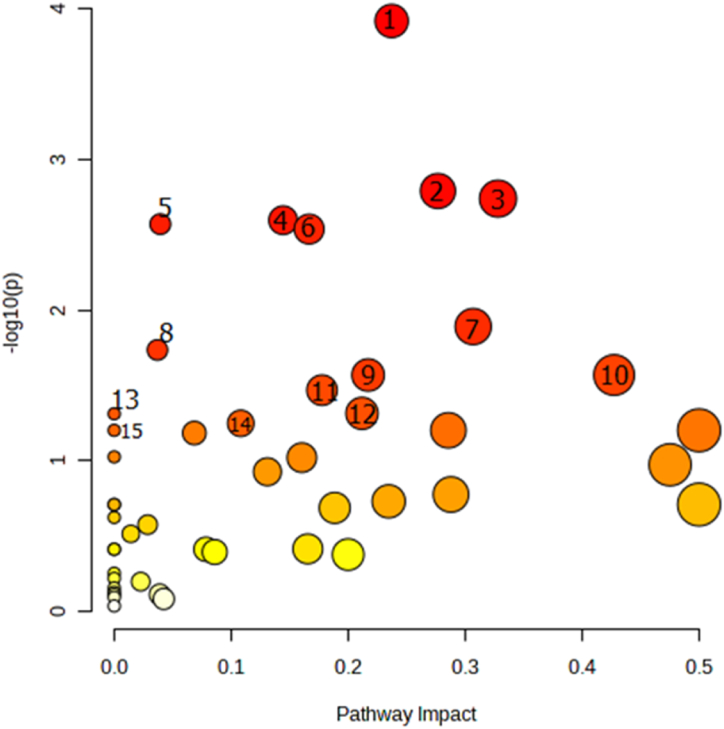
Effects of diabetes and HIIT on Metabolic pathway analysis according to the degree of centrality test.

A pathway analysis was performed, and the results are presented in Table 4, Table 5, Table 6. Fig. 7 shows all the pathways altered in our study according to the degree of centrality.

**Table 4:** Pathway analysis between all groups.

Pathway Name	Total	Hits	P value	-log(p)	FDR	Impact
Pyrimidine metabolism	39	9	1.21E-04	3.9185	0.010134	0.23717
Purine metabolism	65	10	0.0016221	2.7899	0.040641	0.27676
Pentose and glucuronate interconversions	18	5	0.0018228	2.7393	0.040641	0.32812
Amino sugar and nucleotide sugar metabolism	37	7	0.0025355	2.5959	0.040641	0.14442
Glutathione metabolism	28	6	0.0026907	2.5701	0.040641	0.0394
Aminoacyl-tRNA biosynthesis	48	8	0.0029029	2.5372	0.040641	0.16667
Arginine and proline metabolism	38	6	0.012886	1.8899	0.15463	0.30689
Fructose and mannose metabolism	20	4	0.018419	1.7347	0.1934	0.03693
Glycine, serine and threonine metabolism	33	5	0.027014	1.5684	0.22691	0.21707
Cysteine and methionine metabolism	33	5	0.027014	1.5684	0.22691	0.42735
Arginine biosynthesis	14	3	0.034033	1.4681	0.25989	0.17766
Glycerolipid metabolism	16	3	0.048526	1.314	0.30595	0.21183
Thiamine metabolism	7	2	0.048823	1.3114	0.30595	0
Alanine, aspartate and glutamate metabolism	28	4	0.056504	1.2479	0.30595	0.10817
Valine, leucine and isoleucine biosynthesis	8	2	0.0629	1.2013	0.30595	0

Note: Total is the total number of compounds in the pathway; Hits is the matched number from the user-uploaded data; Raw p is the original p value calculated from the enrichment analysis; Holm p is the p-value adjusted by the Holm-Bonferroni method; FDR p is the p-value adjusted using False Discovery Rate; the impact is the pathway impact value calculated from pathway topology analysis.

**Table 5:** Pathway analysis between CTL and DB groups.

Pathway Name	Total	Hits	P value	-log(p)	FDR	Impact
Arginine biosynthesis	14	6	2.71E-05	4.57 E+00	1.92E-03	0.60
Purine metabolism	65	12	4.58E-05	4.34 E+00	1.92E-03	0.22
Alanine, aspartate and glutamate metabolism	28	7	2.89E-04	3.54 E+00	8.10E-03	0.42
Aminoacyl-tRNA biosynthesis	48	9	3.99E-04	3.40 E+00	8.20E-03	0.00
Pentose phosphate pathway	22	6	4.88E-04	3.31 E+00	8.20E-03	0.52
Amino sugar and nucleotide sugar metabolism	37	7	1.75E-03	2.76 E+00	2.45E-02	0.11
Fructose and mannose metabolism	20	5	2.29E-03	2.64 E+00	2.53E-02	0.09
Pyrimidine metabolism	39	7	2.41E-03	2.62 E+00	6.86E-02	0.11
Ascorbate and aldarate metabolism	8	3	5.51E-03	2.26 E+00	6.86E-02	0.50
Galactose metabolism	27	5	9.07E-03	2.04 E+00	5.86E-02	0.13

Note: Total is the total number of compounds in the pathway; Hits is the matched number from the user-uploaded data; p-value calculated from the enrichment analysis; FDR p is the p-value adjusted using the False Discovery Rate; the impact is the pathway impact value calculated from pathway topology analysis.

**Table 6:** Pathway analysis between DB and DTX groups.

Pathway	Total	Hits	P value	-log(p)	FDR	Impact
Arginine biosynthesis	14	8	4.59E-07	6.338	3.85E-05	0.59
Pyrimidine metabolism	39	11	1.55E-05	4.808	6.53E-04	0.32
Aminoacyl-tRNA biosynthesis	48	12	2.41E-05	4.618	6.74E-04	0
Alanine, aspartate and glutamate metabolism	28	8	2.24E-04	3.650	0.0046952	0.64
Arginine and proline metabolism	38	9	4.24E-04	3.372	0.007128	0.37
Pentose and glucuronate interconversions	18	6	5.79E-04	3.237	0.0081	0.5
Valine, leucine and isoleucine biosynthesis	8	4	9.00E-04	3.046	0.0094449	0
Taurine and hypotaurine metabolism	8	4	9.00E-04	3.046	0.0094449	0.71
beta-Alanine metabolism	21	6	1.44E-03	2.840	1.35E-02	0.16
Histidine Metabolism	16	5	2.39E-03	1.790	2.00E-02	0.36

Note: Total is the total number of compounds in the pathway; the Hits is the matched number from the user-uploaded data; p-value calculated from the enrichment analysis; the FDR p is the p-value adjusted using False Discovery Rate; the impact is the pathway impact value calculated from pathway topology analysis.

1-Pyrimidine metabolism, 2- Purine metabolism, 3- Pentose and glucuronate interconversions, 4- Amino sugar and nucleotide sugar metabolism,5- Glutathione metabolism,6- Aminoacyl-tRNA biosynthesis,7- Arginine and proline metabolism,8- Fructose and mannose metabolism,9- Glycine, serine and threonine metabolism, 10- Cysteine and methionine metabolism,11- Arginine biosynthesis, 12- Glycerolipid metabolism, 13- Thiamine metabolism, 14- Alanine, aspartate and glutamate metabolism, 15- Valine, leucine and isoleucine biosynthesis.

**Fig. 8:**
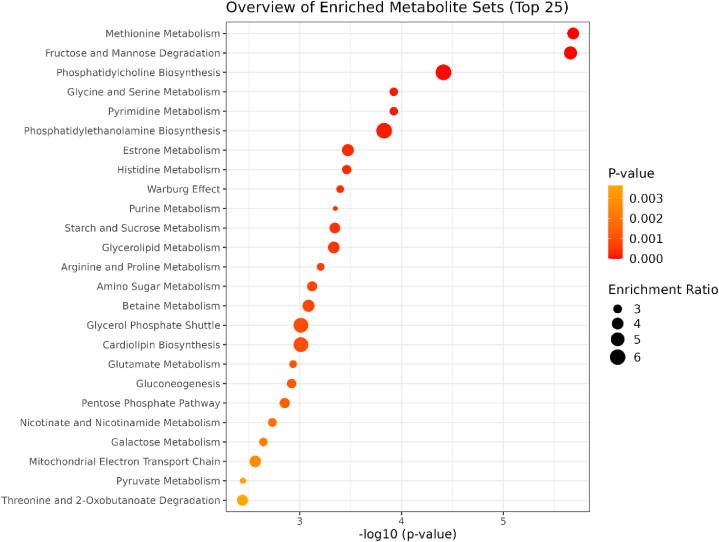
This figure depicts a summary plot for the over-representation analysis (ORA).

**Fig. 9:**
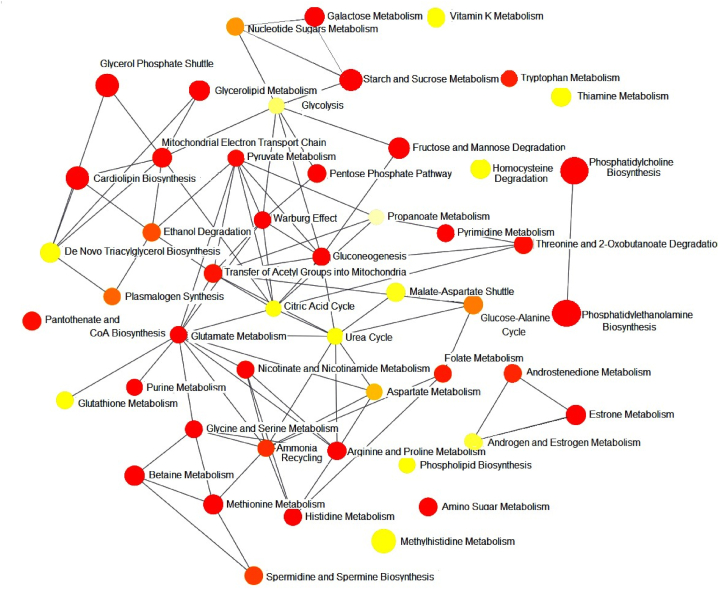
This figure shows the network relationships between the pathways.

Metabolite Set Enrichment Analysis is a way to identify biologically meaningful patterns that was significantly enriched in the quantitative metabolomic data. Overrepresentation analysis was implemented using the hypergeometric test to evaluate whether a particular metabolite set was represented more than expected by chance within the given compound list. One-tailed p-values were obtained after adjusting for multiple tests. Fig. 8 summarizes the results, and Table 7 shows the details of the enrichment pathway analysis. The pathway-related network is illustrated in Fig. 9.

**Table 7:** Result from over-representation analysis.

Metabolite Set	Total	Hits	P value	Holm P	FDR
Methionine Metabolism	43	14	2.06E-06	2.02E-04	1.07E-04
Fructose and Mannose Degradation	32	12	2.19E-06	2.12E-04	1.07E-04
Phosphatidylcholine Biosynthesis	14	7	3.87E-05	0.00371	0.00126
Glycine and Serine Metabolism	59	14	1.19E-04	0.0113	0.00234
Pyrimidine Metabolism	59	14	1.19E-04	0.0113	0.00234
Phosphatidylethanolamine Biosynthesis	12	6	1.48E-04	0.0138	0.00242
Estrone Metabolism	24	8	3.36E-04	0.0309	0.00377
Histidine Metabolism	43	11	3.45E-04	0.0314	0.00377
Warburg Effect	58	13	4.00E-04	0.036	0.00377
Purine Metabolism	74	15	4.46E-04	0.0397	0.00377
Starch and Sucrose Metabolism	31	9	4.51E-04	0.0397	0.00377
Glycerolipids Metabolism	25	8	4.62E-04	0.0402	0.00377
Arginine and Proline Metabolism	53	12	6.22E-04	0.0535	0.00469
Amino Sugar Metabolism	33	9	7.54E-04	0.0641	0.00527
Betaine Metabolism	21	7	8.18E-04	0.0687	0.00534

Note: Total is the total number of compounds in the pathway; Hits is the matched number from the user-uploaded data; p-value calculated from the enrichment analysis; Holm p is the p-value adjusted by the Holm-Bonferroni method; FDR p is the p-value adjusted using False Discovery Rate.

Node size and colors from dark red to pale yellow show the degree of pathway effectiveness in the metabolome profile.

## 4 Discussion

In the present study, we investigated the effect of HIIT on muscle metabolites of T2D rats using metabolomics. The results revealed significant changes in muscle metabolism in T2D rats. Furthermore, HIIT significantly affected muscle metabolism levels in T2D rats. FBG levels increased in the DB group after diabetes induction and decreased after the exercise period in the DB + Ex group compared with those in the DB group (P < 0.05). Homeostatic HOMA-IR was higher in DB compared to CTL and DB + EX (P < 0.05). The essential affected pathways in the DB and EX groups compared with the CTL group and DB + EX group compared with the DB group are discussed here.

### 4.1 Pyrimidine and purine metabolism

Purines and pyrimidines may be synthesized de novo or recycled by normal catabolism via a salvage pathway. The end product of the complete catabolism of purines is uric acid; the catabolism of pyrimidines produces citric acid cycle intermediates [42]. Our results showed that the metabolism of these amino acids was affected by T2D in muscles. Consistent with our results, it has been shown that the metabolism of these amino acids is altered in the eye and urine owing to diabetic retinopathy and diabetic nephropathy, respectively [43,44]. It was also demonstrated that purine metabolites were consistently upregulated in the urinary metabolome of patients diagnosed with gestational diabetes mellitus throughout pregnancy [45]. On the other hand, we showed in our previous study that HIIT could modulate the purinergic signaling in rats with T2D through ATP-dependent channels such as Panax1, P2X7R, and NLRP1 as inflammasome receptors [36]. Our results also confirmed this effect.

Purine metabolism has been shown to play an important role in reducing HOMA-IR and insulin resistance following exercise [46].

### 4.2 Pentose phosphate pathway

The pentose phosphate pathway (the phosphogluconate pathway, hexose monophosphate shunt, or HMP shunt) is a metabolic pathway parallel to glycolysis.

Some studies have shown that this pathway is involved in diabetic retinopathy [47,48].

In line with our results, another study showed that this pathway is altered in skeletal muscles after strenuous exercise [49]. Exercise timing has also been shown to influence this pathway in the skeletal muscles of patients with type 2 diabetes [50].

### 4.3 Amino sugar and nucleotide sugar metabolism

Amino and nucleotide sugar metabolism are closely related to T2D [51]. It has been shown that the levels of *N*-acetyl-d-glucosamine are elevated in T2D, while the levels of l-fucose are reduced due to T2D [52]. The effect of HIIT on these amino acids has yet to be studied.

### 4.4 Glutathione metabolism

Glutathione plays essential roles in antioxidant defense, nutrient metabolism, and regulation of cellular events. Our study found that the glutathione levels were reduced in T2D rats and significantly increased after HIIT. Glutathione is involved in glucose-induced insulin secretion, and the GSH to glutathione disulfide (GSSG) ratio reflects cellular redox homeostasis. In a diabetic rat model, the GSH/GSSG ratio was reduced, indicating an impaired renal glutathione defense system [53]. The GSH/GSSG ratio in the plasma affects cellular responsiveness to glucose, and an increase in this ratio improves the action of peripheral insulin, reduces the extent of oxidative damage, and increases insulin sensitivity in patients with diabetes [54]. We consider this one of the most effective reasons for reducing the FBG and HOMIR levels. In addition, Yu et al. demonstrated that glutathione levels were reduced in T2D rats [52]. Furthermore, strenuous exercise can alter this pathway [55].

### 4.5 Aminoacyl-tRNA biosynthesis pathway

This pathway is pivotal for determining how the genetic code is interpreted as an amino acid [56]. Our results showed that this pathway was affected by both T2D and HIIT and was also affected in the Db + Ex group. Soldado et al. indicated reduced expression of aminoacyl-tRNA synthetases in the skeletal muscle of T2D patients, which may participate in the reduced expression of proteins synthesized in the mitochondria [57,58]. In addition, Tabone et al. revealed that the aminoacyl-tRNA biosynthesis pathway was the most relevant modified pathway in the serum of endurance athletes following acute moderate-intensity exercise [59], which could result from accelerating protein formation in response to exercise. This finding could also explain the activity of the aminoacyl-tRNA biosynthesis pathway observed in this study.

### 4.6 Arginine biosynthesis pathway

This pathway converts glutamate to ornithine and synthesizes arginine from ornithine through the sequential action of enzymes [60]. It has been shown that this pathway can be influenced by diabetic retinopathy [61], and it can be modified in the serum and feces of diabetic monkeys [62]. One study showed that this pathway is upregulated in women with T2D [63]. Some studies have shown that moderate-intensity [64] and aerobic exercise [59] can modulate this pathway. In addition, Zagatto et al. demonstrated the impact of high-intensity exercise on the arginine pathway in human skeletal muscles [65]. They showed that, in addition to the classical expected contribution of glycolytic and phosphagen energetic pathways, high-intensity exercise is also associated with pathways indicative of amino acid and fatty acid oxidation metabolism. Our results also show that DB and EX modified the arginine biosynthesis pathway. However, we did not observe any changes in the DB + EX group compared with the DB group.

### 4.7 Glycine, serine, and threonine metabolism

Serine is derived from 3-phospho-d-glycerate, an intermediate of glycolysis, and glycine is derived from serine. Threonine is an essential amino acid that cannot be synthesized by animals. In a controlled trial, serum analysis by Liu et al. showed that this pathway contributes to gestational diabetes mellitus [66]. Tian et al. showed that this pathway is closely related to renal function in diabetic rats [62]. In another study conducted in Iran, the authors demonstrated the importance of this pathway in diabetic patients [67]. Miao et al. revealed that this pathway could be affected by exhausting swimming training in the serum of mice [68]. Our results showed that EX modified this pathway following DB in the DB + EX group.

### 4.8 Cysteine and methionine metabolism

Cysteine is metabolized to pyruvate via multiple routes. Methionine is an essential amino acid that cannot be synthesized by animals. Cysteine is converted to methionine via cystathionine [69]. Free sulfur amino acids, especially cysteine and methionine, are important in skeletal muscles. However, their concentrations as protein constituents are lower in skeletal muscles than in tissues. Skeletal muscles require both thiol and amino acids. It has been suggested that high levels of cysteine and homocysteine in diabetes are biomarkers of microvascular complications such as diabetic neuropathy, retinopathy, and nephropathy [70].

It has been shown that this pathway can be affected by T2D in the serum of diabetic patients [71]. Another metabolomics study in T2D patients revealed that changes in this pathway following diabetes are inevitable [72]. Disturbed cysteine and methionine metabolism following T2D-induced nephropathy has been reported by Peng et al. [73]. In addition, serum metabolomics revealed a potential benefit of methionine in patients with type 1 diabetes with poor glycemic control and high glycemic variability [74].

Exhaustive exercise has been demonstrated to affect plasma and urine concentrations of sulfur amino acids, such as cysteine, and the ratio of methionine to homocysteine [75]. One study reported that acute exercise alters homocysteine plasma concentration in an intensity-dependent manner owing to increased methyl flux in the livers of rats [76]. Methionine is one of the metabolites that can affect blood glucose following exercise [77].

Our results also showed changes in these amino acids owing to diabetes and HIIT.

### 4.9 Thiamine metabolism

Thiamin (thiamine), or vitamin B1, is a water-soluble vitamin found naturally in some foods, added to foods, and sold as a supplement. Thiamin plays a vital role in the growth and function of various cells.

Some studies revealed thiamine metabolism changes in the serum of patients with diabetic retinopathy [78,79]. Thiamine metabolism was enriched in this study, mainly because of the significantly reduced thiamine triphosphate and elevated l-cysteine levels in diabetes. Thiamine triphosphate is the non-coenzyme form of vitamin B1 found in all living organisms. Although the mechanism is currently unclear, thiamine triphosphate is considered to be the allosteric activator of glutamate dehydrogenase, which promotes the metabolism of glutamate to alpha-ketoglutarate (essential substances for the tricarboxylic acid cycle) [80]. Some investigations disclosed that exercise has an important impact on thiamine metabolism in the blood [81,82]. Our results were consistent with those of the studies above.

### 4.10 Alanine, aspartate, and glutamate metabolism

Glutamate, aspartate, asparagine, l-alanine, and d-alanine stem from intermediates found within central metabolism, primarily within the citric acid cycle, often through one or two sequential steps. Despite the brevity of these pathways, the significance and intricacy of the roles of these amino acids align with their close connection to central metabolic processes. The metabolism of alanine, aspartate, and glutamate is affected by T2D. Changes in the metabolism of these amino acids have been reported in some studies on diabetic retinopathy [43,83,84]. Our findings revealed that HIIT did not affect galactose metabolism.

Alanine, glutamate, and carnitine show higher plasma concentrations in T2D during exercise or recovery and can affect blood glucose levels [85]. On the other hand, aspartate metabolism is important in reducing insulin resistance following exercise [86].

### 4.11 Valine, leucine, and isoleucine biosynthesis pathway

The valine biosynthesis pathway is a four-step pathway that shares all its steps with the parallel isoleucine biosynthesis pathway. These entwined pathways are part of the super pathway of branched-chain amino acid biosynthesis that generates isoleucine, valine, and leucine. Alterations in the valine, leucine, and isoleucine biosynthesis pathways have been reported in diabetic retinopathy in the vitreous and aqueous humor [87] and diabetic nephropathy in the urine [72]. Del Coco et al. revealed that this pathway was modified in the serum of T2D patients [88]. Resistance training has been shown to modulate this pathway in the hippocampus of aged rats [89]. Our results revealed that this pathway was modified in the diabetes/exercise group compared with the diabetes group. Valine, leucine, and isoleucine are brain-chain amino acids, the only amino acids used in skeletal muscle during exercise [90]. As HIIT is a high-demand exercise, it may increase the turnover of these amino acids. However, valine and leucine metabolism significantly reduce insulin resistance following exercise [91].

## 5 Conclusion

**Fig. 10:**
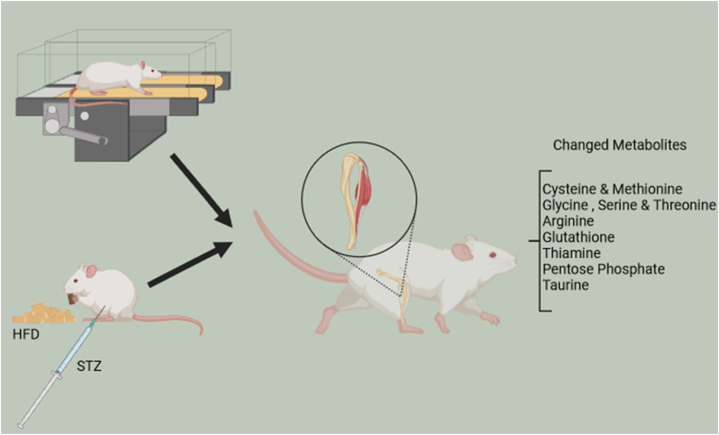
Metabolites affected by T2D and HIIT in muscle tissue.

In conclusion, our study identified several metabolic alterations in different pathways in the muscles of diabetic rats and explored the modifications generated by HIIT. These findings may provide novel insights into the pathophysiology of T2D and the mechanisms by which HIIT can help improve T2D muscle complications. These data may help develop preventive strategies and effective treatments (Fig. 10).

### 5.1 Study limitation and perspective for future studies

We used 400 MHz One-dimensional 1H NMR, which might limit metabolite detection. However, future studies may use liquid chromatography-mass spectrometry (LC-MS) or high-resolution HNMR. Furthermore, investigating the effect of various HIIT protocols using metabolomics could help us suggest the most effective protocol for improving IR in patients with diabetes.

Increasing the sample size in future studies will also help to have a more precise conclusion since, due to the mortality caused by diabetes and compliance with ethical principles, we had a limited sample size.

## Data availability

All data are available upon request.

## Ethics approval declaration

This study was reviewed and approved by the ethics committee of Kerman University of Medical Sciences with the approval number: IR. KMU.REC.1399.688, approval on 10/3/2021.

## CRediT authorship contribution statement

**Kayvan Khoramipour:** Investigation, Conceptualization. **Mohammad Amin Rajizadeh:** Writing – original draft. **Ziba Akbari:** Investigation. **Mohammad Arjmand:** Methodology.

## Declaration of competing interest

The authors declare the following financial interests/personal relationships which may be considered as potential competing interests:Mohammad Arjmand reports was provided by Pasteur Institute of Iran. Mohammad Arjmand reports a relationship with Pasteur Institute of Iran that includes: board membership. Mohammad Arjmand has patent pending to N/A. The authors declare no competing of interest. If there are other authors, they declare that they have no known competing financial interests or personal relationships that could have appeared to influence the work reported in this paper.
